# The potential biomarker value of soluble CD36 in the treatment of diabetic kidney disease: evidence from GLP-1 and insulin interventions

**DOI:** 10.3389/fendo.2025.1605631

**Published:** 2025-05-27

**Authors:** Wenxuan Li, Yangang Wang

**Affiliations:** Department of Endocrinology, The First Affiliated Hospital of Qingdao University, Qingdao, China

**Keywords:** DKD, soluble CD36, GLP-1RAs, biomarker, urinary albumin-to-creatinine ratio

## Abstract

**Background:**

Soluble CD36 (sCD36), the circulating form of the scavenger receptor CD36, plays a key role in lipid accumulation and inflammation during the progression of diabetic kidney disease (DKD), and has been proposed as a promising non-invasive biomarker. The renoprotective effects of glucagon-like peptide-1 receptor agonists (GLP-1RAs) may involve modulation of sCD36. This study aimed to evaluate the impact of GLP-1RA and insulin treatment on sCD36 levels and their association with renal function in DKD patients.

**Methods:**

This single-center, prospective observational cohort study enrolled 191 patients with type 2 diabetes and early-stage DKD, who were stratified into three groups based on treatment regimen: control group (n = 63), insulin group (n = 71), and GLP-1RA group (n = 57). All patients received standard care with metformin, with the insulin and GLP-1RA groups receiving additional respective treatments for 12 weeks. Clinical parameters including sCD36, urinary albumin-to-creatinine ratio (UACR), lipid profile, glycemic markers, and islet function indices were assessed at baseline and post-treatment. Intra- and inter-group comparisons were performed using paired tests and analysis of covariance. Generalized linear regression models were applied to assess the relationship between sCD36 and renal function.

**Results:**

Baseline sCD36 and UACR levels were comparable across the three groups (*P > 0.05*). After 12 weeks, sCD36 levels significantly declined in the GLP-1RA group (median: 195.20 ng/mL, IQR: 160.45–314.75), compared to the insulin group (364.60 ng/mL, IQR: 279.10–394.10) and control group (386.10 ng/mL, IQR: 323.60–471.30) (*P < 0.001*). The GLP-1RA group also showed the most marked reduction in UACR (*P < 0.001*). Regression analysis demonstrated a significant positive association between sCD36 and UACR levels both before and after treatment (*P < 0.001*), and the change in sCD36 (ΔsCD36) was positively correlated with the improvement in UACR, suggesting a link to reduced renal lipotoxicity and inflammation.

**Conclusion:**

GLP-1RAs significantly reduce sCD36 and UACR levels in patients with early DKD, outperforming insulin in renoprotection. These findings raise the possibility that GLP-1RAs may exert renoprotective effects through modulation of CD36-related pathways, although direct mechanistic validation was not performed in this study.sCD36 may serve as a useful biomarker for monitoring DKD progression and therapeutic response, though further multicenter and long-term studies are needed to confirm its clinical utility.

## 1 Introduction

Diabetic kidney disease (DKD) is one of the most common microvascular complications of diabetes and a leading cause of end-stage renal disease (ESRD) worldwide. Epidemiological data show that approximately 20% to 40% of individuals with diabetes eventually develop DKD, with its incidence rising in parallel with the global diabetes epidemic (1, 2). Despite advances in glycemic control and therapeutic interventions, the burden of DKD remains substantial, highlighting the need for deeper insights into its pathophysiology and more effective intervention strategies.

Recent research has shed light on lipotoxicity and inflammation as central mechanisms in DKD progression, and CD36, a class B scavenger receptor and fatty acid translocase, has emerged as a key molecule in this context. CD36 is highly expressed in renal tissues of patients with DKD and contributes to tubular lipid accumulation, mitochondrial dysfunction, and activation of pro-inflammatory pathways (3, 4). Mechanistically, CD36 promotes fatty acid uptake while inhibiting mitochondrial fatty acid oxidation (FAO), leading to excess production of mitochondrial reactive oxygen species (mtROS) and activation of the NLRP3 inflammasome (5, 6). It may also exacerbate tubulointerstitial fibrosis through modulation of the Wnt/β-catenin signaling pathway (7). Of particular interest is soluble CD36 (sCD36), a circulating form of the receptor released via proteolytic cleavage. Serum sCD36 levels have been correlated with tissue CD36 expression and are elevated in patients with type 2 diabetes mellitus (T2DM), especially those with lipid-induced renal injury and inflammation (7, 8), suggesting its potential as a non-invasive biomarker of DKD progression.

Although the role of CD36 in DKD has been increasingly recognized in preclinical studies, few clinical investigations have examined how pharmacological interventions affect circulating sCD36 levels in DKD patients. In particular, glucagon-like peptide-1 receptor agonists (GLP-1RAs) are of growing interest due to their ability to modulate lipid metabolism and exert anti-inflammatory and renoprotective effects. Preclinical data indicate that GLP-1RAs may reduce CD36 expression in renal tissues, leading to decreased fatty acid uptake and lipid accumulation, and ultimately improving renal outcomes (9, 10). However, whether GLP-1RA treatment influences circulating sCD36 levels in patients with DKD, and how these changes correlate with renal function indicators such as urinary albumin-to-creatinine ratio (UACR), remains poorly understood.

To address this gap, the present study was designed to evaluate the comparative effects of GLP-1RA and insulin therapy on sCD36 levels in patients with early-stage DKD using real-world clinical data. Furthermore, we aimed to assess the association between sCD36 levels and UACR to explore the potential of sCD36 as a dynamic biomarker reflecting therapeutic responses and disease progression. Our study represents one of the first prospective clinical investigations to systematically evaluate sCD36 changes under GLP-1RA intervention in DKD patients, thereby contributing novel clinical evidence to support its biomarker potential and therapeutic relevance.

## 2 Subjects and methods

### 2.1 Study design

This study was designed as a real-world, prospective, observational cohort study conducted at the Affiliated Hospital of Qingdao University. Patients with type 2 diabetes mellitus (T2DM) who were hospitalized in the Department of Endocrinology and Metabolic Diseases between 2024 and 2025 were screened for eligibility. All participants met the predefined inclusion and exclusion criteria and provided written informed consent prior to enrollment. The study protocol was approved by the Medical Ethics Committee of the Affiliated Hospital of Qingdao University (Ethical Approval No.QYFYWZLL29898).To minimize selection bias, an stratified assignment strategy was employed. Eligible patients were stratified and assigned in a 1:1:1 ratio to the control group, the GLP-1RA group, or the insulin group using a computer-generated block randomization system, managed by a research assistant who was not involved in clinical treatment or data collection. The allocation sequence was blinded to the primary investigators, thereby enhancing objectivity and comparability between groups. Although this was an observational study and not a randomized controlled trial (RCT), the use of structured allocation procedures strengthened the internal validity of the group comparisons. The study was conducted in strict accordance with the principles of Good Clinical Practice (GCP), the Declaration of Helsinki, and relevant Chinese regulations on clinical research ethics. All participants provided written informed consent. Baseline demographic data (e.g., height, weight, age) were collected by trained healthcare professionals and kept strictly confidential. All patient-related information was used solely for research purposes and was not disclosed or repurposed without authorization. Sodium-glucose co-transporter-2 (SGLT2) inhibitors were excluded from this study to avoid confounding effects on renal outcomes, given their known nephroprotective properties and potential influence on inflammation and oxidative stress. This also allowed a more focused comparison between GLP-1 receptor agonists and insulin with respect to their impact on sCD36 and UACR.

**Figure 1:**
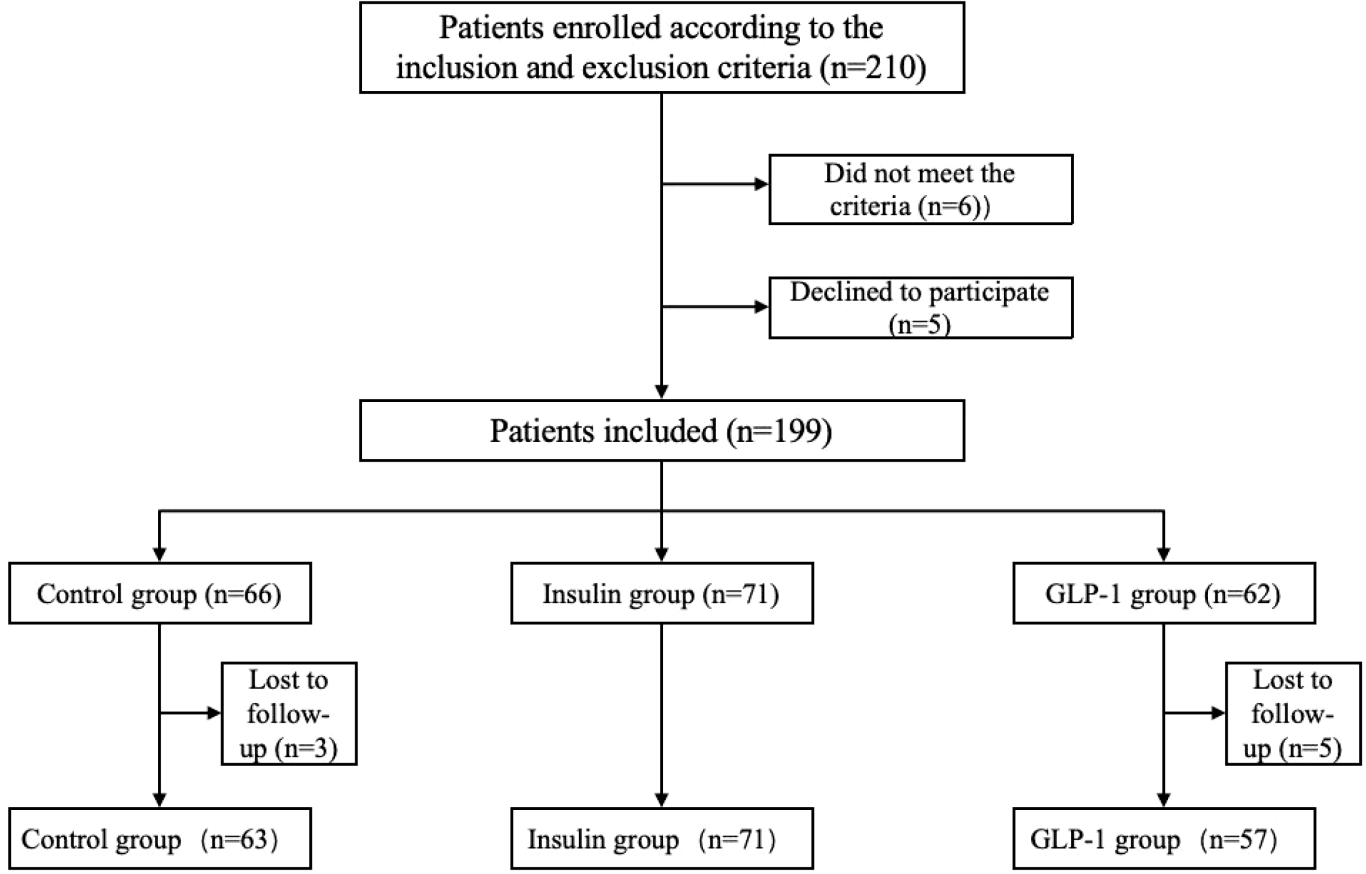
Flowchart.

Based on *a priori* power analysis using G*Power (effect size: Cohen’s d = 0.8; α = 0.05; power = 80%), a minimum of 70 participants per group was required to achieve statistical significance. A total of 191 patients were ultimately enrolled in this study (**Figure 1**), meeting the criteria for valid statistical analysis. Participants were screened according to predefined inclusion and exclusion criteria and assigned to one of three treatment groups based on their therapeutic regimen: the control group, the GLP-1RA group, and the insulin group. All participants received a 12-week course of treatment. Metformin (500 mg, twice daily) was administered as background therapy in all groups. The control group received no additional intervention. The GLP-1RA group received subcutaneous injections of a GLP-1 receptor agonist (0.5 mg weekly) in addition to metformin. The insulin group received daily subcutaneous insulin injections, with dosages adjusted individually based on glycemic status.

Data Collection: At baseline and after 12 weeks of treatment, fasting venous blood samples were collected to measure the following clinical indicators: triglycerides (TG), total cholesterol (TC), low-density lipoprotein cholesterol (LDL-C), high-density lipoprotein cholesterol (HDL-C), free fatty acids (FFA), glycated hemoglobin (HbA1c), fasting insulin, fasting C-peptide, sCD36, urinary albumin-to-creatinine ratio (UACR), alanine aminotransferase (ALT), aspartate aminotransferase (AST), and serum uric acid. Blood was collected into procoagulant separation tubes (≥1 mL), stored at 4°C, and centrifuged within 2 hours at 3000 rpm for 10 minutes. The supernatant was aliquoted and stored at –80°C for subsequent analysis. Serum sCD36 concentrations were measured using a commercially available enzyme-linked immunosorbent assay (ELISA) kit, following the manufacturer’s protocol. All samples were analyzed in the same experimental batch. Laboratory staff were blinded to group assignments, and the inter-assay variability was controlled within 10%.

Statistical Analysis: Changes in key clinical indicators before and after treatment were compared within and between groups to evaluate the efficacy of different therapeutic regimens in glycemic control, lipid regulation, renal protection, and modulation of sCD36 levels. In addition, intergroup comparisons were performed, and adverse events were recorded to assess the overall safety and clinical utility of each intervention.

### 2.2 Study population

Inclusion Criteria: Participants were eligible for inclusion if they met all of the following criteria:1. Age between 15 and 75 years;2. Body mass index (BMI) ranging from 18.5 to 40 kg/m²;3. Poor glycemic control, defined as glycated hemoglobin A1c (HbA1c) levels between 7.5% and 10% despite adherence to diet and exercise interventions;4. Urinary albumin-to-creatinine ratio (UACR) between 30 and 300 mg/g, indicating microalbuminuria;5. No prior use of GLP-1RAs or insulin for glycemic management.

Exclusion Criteria: Participants were excluded from the study if they met any of the following criteria:1. History of acute diabetic complications such as diabetic ketoacidosis (DKA) or hyperosmolar hyperglycemic syndrome (HHS);2. Presence of treatment-resistant hypertension requiring four or more antihypertensive agents for control;3. Recent acute cardiovascular or cerebrovascular events, active infections, ketoacidosis, stress-related conditions, hepatic or renal dysfunction, anemia, other endocrine disorders, primary acute or chronic glomerulonephritis, urinary tract infections, urinary obstruction, or other urinary system diseases;4. Use of glucocorticoids or other medications that affect blood glucose levels, or use of diuretics and other drugs that may interfere with urinary albumin excretion rate (UAER) measurements;5. Presence of cardiac pacemakers, neurostimulators, or metallic prosthetic heart valves;6. Comorbid hepatic-pancreatic-renal conditions, including liver cirrhosis, active hepatitis, or impaired renal function (estimated glomerular filtration rate, eGFR < 60 mL/min/1.73 m²);7. History of malignancy or other serious illnesses within the past five years at the time of screening.

### 2.3 Statistical analysis

All statistical analyses were performed using IBM SPSS Statistics version 29.0 (IBM Corp., Armonk, NY, USA). Continuous variables were expressed as mean ± standard deviation (SD). For normally distributed data, paired t-tests were used to compare pre- and post-treatment values. For non-normally distributed data, the Wilcoxon signed-rank test was applied. Comparisons among the three groups were conducted using one-way analysis of variance (ANOVA), ANCOVA was employed to adjust for significant baseline differences in HbA1c and other metabolic parameters. These variables were entered as covariates to reduce potential bias in the estimation of treatment effects. When statistical significance was observed, the least significant difference (LSD) test was used for *post hoc* pairwise comparisons. For non-normally distributed variables, the Kruskal–Wallis test was employed, followed by Dunn–Bonferroni *post hoc* correction for multiple comparisons. Categorical variables were expressed as frequencies and percentages and compared using the chi-square test or Fisher’s exact test as appropriate.

### 2.4 Comparison of baseline characteristics

Baseline characteristics among the three groups (control, GLP-1RA, and insulin groups) were compared using one-way analysis of variance (ANOVA) for normally distributed continuous variables, the Kruskal–Wallis test for non-normally distributed variables, and the chi-square (χ²) test for categorical variables. Within-group changes in metabolic parameters before and after treatment were evaluated using paired t-tests for normally distributed variables or the Wilcoxon signed-rank test for non-normally distributed variables. Key metabolic parameters analyzed included triglycerides (TG), total cholesterol (TC), low-density lipoprotein (LDL), high-density lipoprotein (HDL), free fatty acids (FFA), HbA1c, fasting insulin, fasting C-peptide, sCD36, urinary albumin-to-creatinine ratio (UACR), alanine aminotransferase (ALT), aspartate aminotransferase (AST), and serum uric acid. Between-group comparisons of changes in these metabolic and biochemical parameters after treatment were conducted using ANOVA or the Kruskal–Wallis test, as appropriate. To control for potential pre-treatment differences, analysis of covariance (ANCOVA) was applied, followed by *post hoc* testing using the least significant difference (LSD) method to identify pairwise group differences.

### 2.5 Analysis of specific indicators

sCD36, the circulating form of CD36, is typically released into the bloodstream via proteolytic cleavage from tissue-expressed CD36. Measurement of serum sCD36 levels provides a non-invasive approach to indirectly assess CD36 activity in renal tissue. The following analyses were performed: Group Comparisons: Changes in sCD36 levels before and after treatment were compared among the control, GLP-1RA, and insulin groups using analysis of variance (ANOVA) or the Kruskal–Wallis test, depending on data distribution. *Post hoc* comparisons between groups were conducted using Bonferroni correction to identify statistically significant pairwise differences.GLP-1RA–Specific Analysis: In patients treated with GLP-1RAs, the change in sCD36 levels before and after treatment (ΔsCD36) was calculated. The relationship between ΔsCD36 and improvements in renal function, as reflected by changes in UACR, was further examined. Correlation and Regression: Correlations between sCD36 levels and renal function indicators, such as UACR and estimated glomerular filtration rate (eGFR), were evaluated using either Spearman or Pearson correlation analysis, depending on variable distribution. To assess whether sCD36 was independently associated with renal function, multivariable linear or logistic regression models were employed, adjusting for potential confounders including age, sex, body mass index (BMI), and duration of diabetes. Subgroup Analysis and Comparative Evaluation: Subgroup comparisons were performed to assess the magnitude of change in sCD36, UACR, and other core indicators. Differences in therapeutic effects between the GLP-1RA and insulin groups were also analyzed to evaluate the relative impact of each intervention on sCD36 modulation and renal function outcomes.

### 2.6 Results analysis

Statistical significance was defined as p < 0.05 (*), and highly significant results were indicated as p < 0.01 (**). To enhance the reliability of the findings, all tests were two-tailed, with a significance threshold set at 5%. For variables demonstrating significant correlations, partial correlation analyses were further conducted to account for the influence of potential confounding factors.

## 3 Results

### 3.1 Baseline characteristics

A total of 191 participants completed the study and were categorized into three groups according to their treatment regimen: control group (n = 63), insulin group (n = 71), and GLP-1RA group (n = 57). Baseline characteristics for all groups are summarized in **Table 1**. No statistically significant differences were observed among the groups at baseline with respect to body weight, duration of diabetes, lipid parameters (TG, TC, LDL-C, HDL-C, FFA), liver function indicators (AST, ALT), urinary albumin-to-creatinine ratio (UACR), or sCD36 levels (all *p > 0.05*).

**Table 1:** Comparison of general characteristics and baseline clinical parameters of the subjects.

Variable	Control group (N=63)	Insulin group (N=71)	GLP-1 group (N=57)	F/H/χ²	*P*
Age (years)	60.13 ± 10.24	61.00 (50.75~71.00)	52.75 ± 15.24	6.64	*p=0.036*
Height (cm)	168.63 ± 8.05	168.08 ± 8.48	166.16 ± 8.63	1.64	*p=0.197*
Weight (kg)	72.93 ± 11.60	73.50 (66.75~85.00)	76.50 (65.00~87.00)	1.14	*p=0.567*
BMI	25.82 ± 3.14	26.53 (24.37~30.07)	27.18 (25.00~29.83)	8.15	*p=0.017*
Male (%)	63.5	49.3	31.6	12.10	*p<0.001*
Duration of diabetes (years)	10.00 (6.00~20.00)	10.00 (3.00~17.00)	7.50 (4.00~12.75)	3.509	*p=0.173*
FBG (mmoll)	7.47 ± 1.53	9.02 (7.89~10.32)	8.07 ± 1.52	29.531	*p<0.001*
HbA1c (%)	7.75 (6.98~8.70)	9.35 (8.00~11.05)	8.39 ± 1.44	22.667	*p<0.001*
TG (mmol/l)	1.55 (0.94~2.64)	1.76 (1.19~2.68)	1.91 (1.31~3.04)	5.323	*p=0.07*
TC (mmol/l)	4.12 (3.62~5.09)	4.76 ± 1.31	4.75 ± 1.34	5.302	*p=0.071*
LDL-C (mmol/l)	2.35 ± 0.72	2.72 ± 1.02	2.66 ± 1.06	3.20	*p=0.043*
HDL-C (mmol/l)	1.17 ± 0.31	1.10 ± 0.30	1.05 ± 0.25	2.75	*p=0.067*
FFA (mmoll)	0.43 (0.29~0.62)	0.40 (0.28~0.49)	0.48 (0.35~0.60)	3.707	*p=0.157*
UA (umoll)	344.50 (293.75~430.25)	336.45 ± 94.00	350.59 ± 88.88	1.473	*p=0.479*
BUN (umol/l)	6.36 (5.05~8.04)	5.57 (4.56~7.09)	6.01 (4.78~7.27)	5.495	*p=0.064*
Ser (umoll)	66.00 (51.75~90.75)	52.00 (43.00~62.00)	71.45 (54.00~86.60)	24.568	*p<0.001*
eGFR (mL/min/1.73m²)	91.52 ± 18.81	100.92 (94.32~115.56)	91.03 ± 21.74	16.489	*p<0.001*
AST (U/L)	17.00 (14.00~21.00)	17.00 (15~23.00)	19.90 (15.00~29.00)	4.932	*p=0.064*
ALT (U/L))	17.50 (11.75~25.00)	18.50 (15.00~25.25)	21.50 (13.00~46.75)	5.623	*p=0.06*
Fasting insulin (uIUmL)	3.57 (1.89~8.89)	7.57 (4.63~12.63)	12.15 (6.39~18.73)	33.748	*p<0.001*
Fasting C-peptide (ng/ml)	3.23 (2.03~7.90)	2.04 (1.33~2.85)	2.86 ± 1.45	19.085	*p<0.001*
UACR (mg/g)	38.95 (33.22~76.89)	39.15 (35.06~53.62)	39.44 (35.52~48.50)	0.179	*p=0.915*
HOMA-IR	1.25 (0.54~2.99)	3.07 (1.72~5.52)	4.07 (2.24~6.61)	35.057	*p<0.001*
HOMA-β	20.71 (11.08~37.02)	28.09 (16.95~39.99)	56.54 (29.60~91.92)	26.797	*p<0.001*
sCD36	388.50 (342.55~466.10)	384.45 (349.28~440.33)	386.55 (354.85~437.23)	0.187	*p=0.911*

BMI, Body Mass Index; FBG, Fasting Blood Glucose; BUN, Blood Urea Nitrogen; HbA1c, Glycated Hemoglobin; TG, Triglycerides; TC, Total Cholesterol; LDL-C, Low-Density Lipoprotein Cholesterol; HDL-C, High-Density Lipoprotein Cholesterol; FFA, Free Fatty Acids; UA, Uric Acid; Scr, Serum Creatinine; eGFR, Estimated Glomerular Filtration Rate; ALT, Alanine Aminotransferase; AST, Aspartate Aminotransferase; UACR, Urinary Albumin-to-Creatinine Ratio; HOMA-IR, Homeostasis Model Assessment of Insulin Resistance; HOMA-β, Homeostasis Model Assessment of β-cell Function; sCD36, Soluble CD36; DKD, Diabetic Kidney Disease.

### 3.2 Between-group comparison of post-treatment clinical indicators

**Figure 2:**
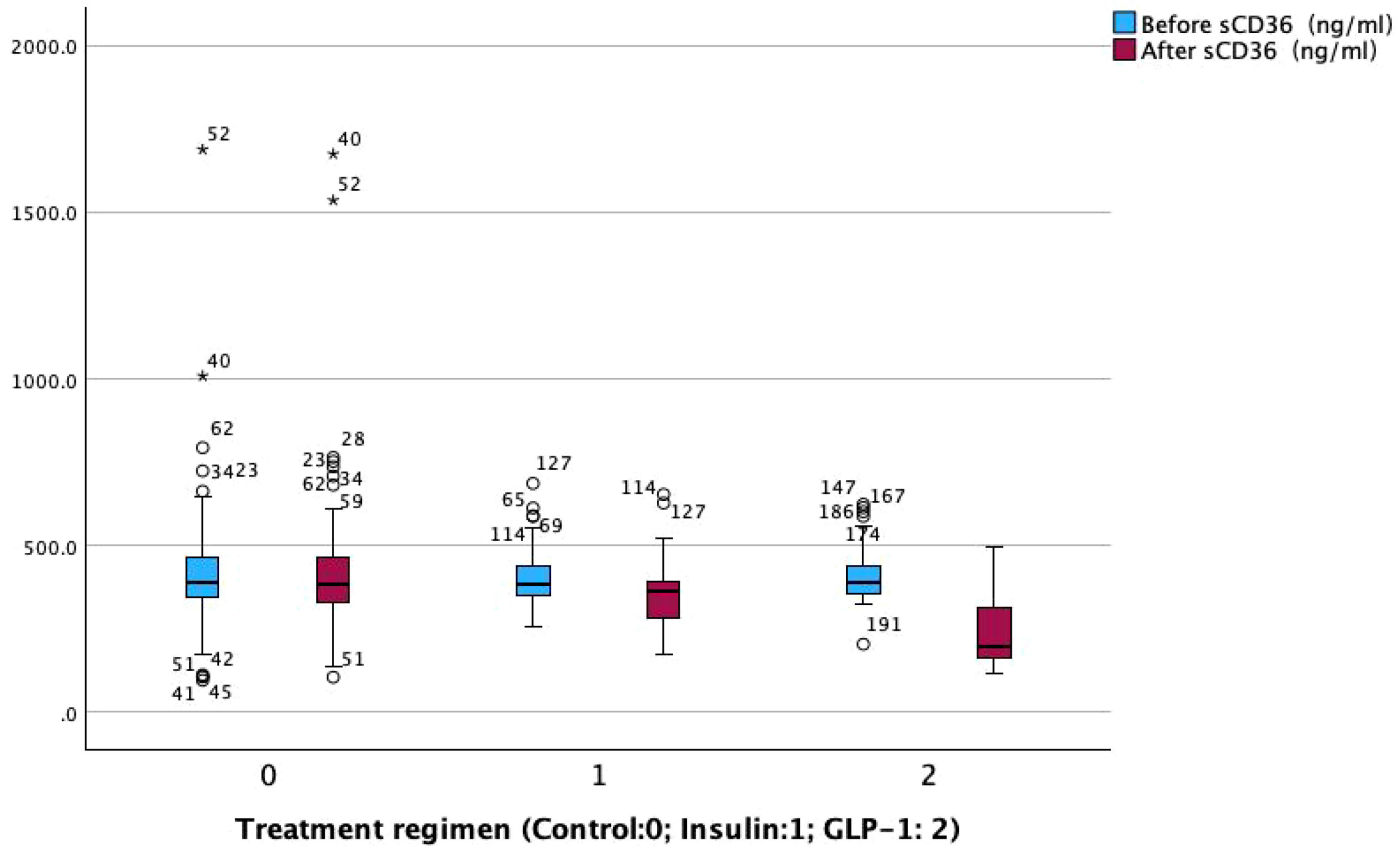
Comparison of soluble CD36 levels before and after treatment among the three groups.

Post-treatment comparisons of clinical parameters among the three groups are presented in **Table 2A**. Significant between-group differences were observed in several metabolic and renal function markers, including fasting blood glucose (FBG), glycated hemoglobin (HbA1c), fasting insulin, fasting C-peptide, homeostasis model assessment of insulin resistance (HOMA-IR), β-cell function index (HOMA-β), urinary albumin-to-creatinine ratio (UACR), and sCD36 levels (all *p < 0.05*; see **Figure 2**).Notably, sCD36 levels were significantly lower in the GLP-1RA group [median: 195.20 ng/mL, interquartile range (IQR): 160.45–314.75], followed by the insulin group [364.60 ng/mL, IQR: 279.10–394.10], with the highest levels observed in the control group [386.10 ng/mL, IQR: 323.60–471.30]. The difference among groups was statistically significant (F = 5.098, *p < 0.05*).In contrast, no significant differences were found among groups for lipid parameters (TG, TC, LDL-C, HDL-C, FFA) or liver function markers (AST, ALT) (*p > 0.05* for all). Combined with baseline findings, where no between-group differences were noted in UACR, sCD36, or lipid/liver function parameters, these results suggest that the observed post-treatment changes in UACR and sCD36 may be attributed to the different therapeutic regimens, whereas changes in lipid and hepatic markers were not statistically significant.

**Table 2A:** Comparison of clinical parameters after treatment. TABLE 2 (a)Between-group analysis of clinical parameters after treatment.

Variable	Control group (N=63)	Insulin group (N=71)	GLP-1 group (N=57)	F/H	*P*
FBG (mmoll)	6.60 ± 1.01	7.03 (6.20~7.75)	7.10 ± 1.05	5.468	*p<0.05*
HbA1c (%)	6.74 ± 0.97	7.16 ± 1.05	6.72 ± 0.90	4.238	*p<0.05*
TG (mmol/l)	1.33 (0.95~1.77)	1.40 (1.12~2.12)	1.37 (1.08~2.05)	1.759	*p=0.185*
TC (mmol/l)	4.08 ± 0.89	4.22 ± 0.99	4.02 ± 0.92	0.77	*p=0.464*
LDL-C (mmol/l)	1.97 (1.28~2.31)	2.08 (1.55~2.87)	2.13 ± 0.76	2.54	*p=0.111*
HDL-C (mmol/l)	1.30 ± 0.30	1.23 (1.05~1.33)	1.20 (1.05~1.32)	1.155	*p=0.283*
FFA (mmoll)	0.36 (0.29~0.44)	0.33 (0.21~0.44)	0.30 (0.24~0.38)	1.435	*p=0.231*
UA (umoll)	306.00 (252.00~382.00)	295.00 (246.00~370.00)	288.00 (222.50~359.50)	1.465	*p=0.226*
AST (U/L)	17.24 ± 4.24	17.00 (14.00~21.00)	17.00 (14.00~21.50)	0.135	*p=0.714*
ALT (U/L))	18.00 (14.00~25.00)	20.00 (16.00~27.00)	21.00 (14.50~34.5)	3.434	*p=0.064*
Fasting insulin (uIUmL)	3.67 (2.14~7.94)	9.51 (5.97~14.35)	13.95 ± 7.57	20.899	*p<0.001*
Fasting C-peptide (ng/ml)	3.94 (1.98~7.61)	2.19 (1.46~3.46)	3.41 ± 1.47	14.732	*p<0.001*
UACR (mg/g)	38.73 (32.51~71.93)	36.37 (27.40~40.68)	20.31 (14.33~32.86)	3.935	*p<0.05*
HOMA-IR	1.15 (0.61~2.32)	3.04 (1.69~4.55)	4.27 (2.64~5.45)	23.545	*p<0.001*
HOMA-β	26.07 (13.01~62.58)	58.24 (33.82~90.03)	78.87 ± 40.52	13.476	*p<0.001*
sCD36	386.10 (323.60~471.30)	364.60 (279.10~394.10)	195.20 (160.45~314.75)	5.098	*p<0.05*

FBG, Fasting Blood Glucose; BUN, Blood Urea Nitrogen; HbA1c, Glycated Hemoglobin; TG, Triglycerides; TC, Total Cholesterol; LDL-C, Low-Density Lipoprotein Cholesterol; HDL-C, High-Density Lipoprotein Cholesterol; FFA, Free Fatty Acids; UA, Uric Acid; ALT, Alanine Aminotransferase; AST, Aspartate Aminotransferase; UACR, Urinary Albumin-to-Creatinine Ratio; HOMA-IR, Homeostasis Model Assessment of Insulin Resistance; HOMA-β, Homeostasis Model Assessment of β-cell Functions; CD36, Soluble CD36.

### 3.3 Within-group paired analyses of treatment effects

**Figure 3:**
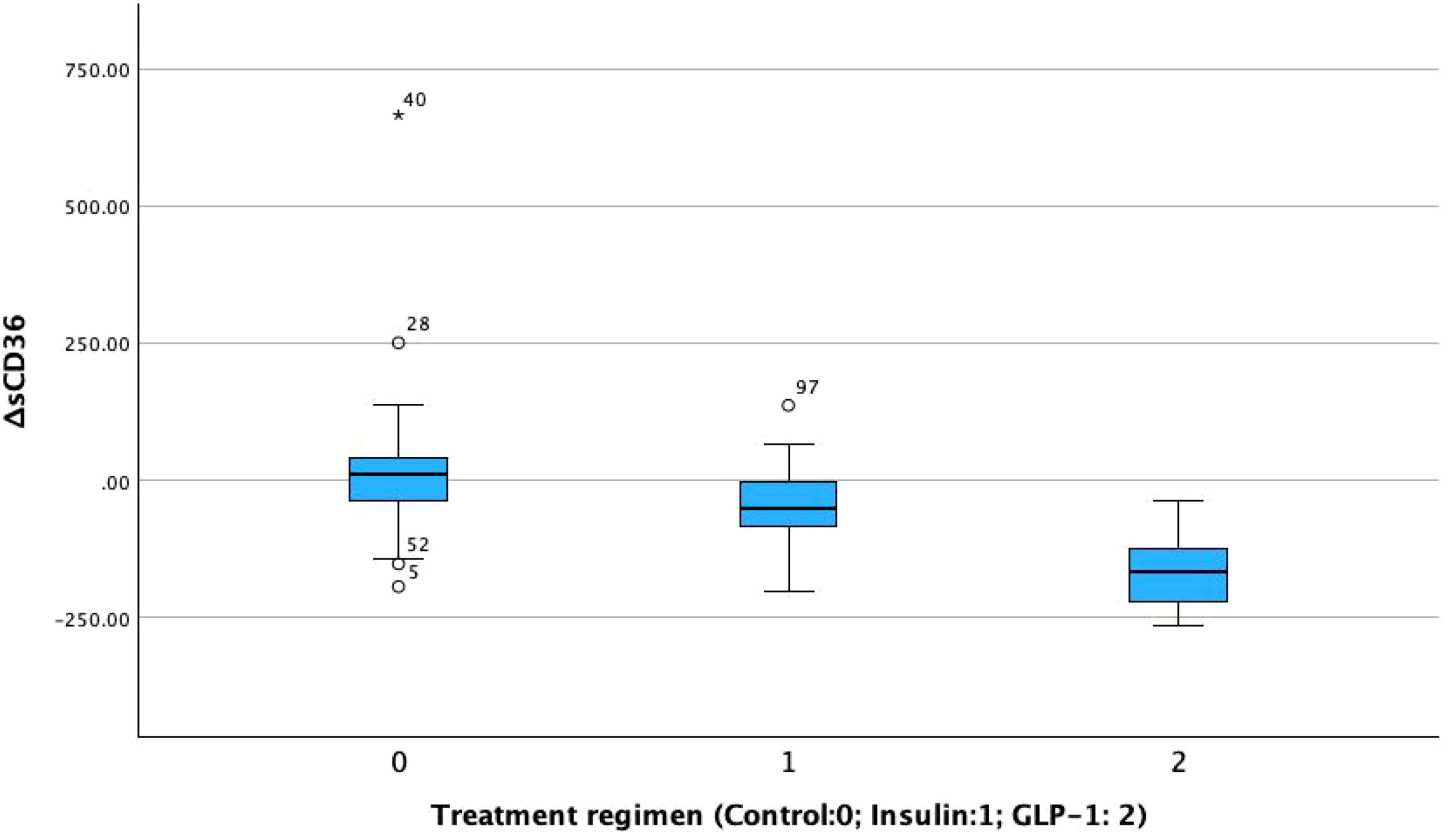
Comparison of differences in soluble CD36 levels.

To further evaluate the effects of different therapeutic strategies, within-group comparisons were performed for key clinical parameters before and after treatment (**Table 2B**). Significant improvements were observed in glycemic control and lipid metabolism across all three groups following treatment (*p < 0.05*).Regarding liver function, both AST and ALT levels significantly decreased in the GLP-1RA group (AST: *p < 0.001*; ALT: *p < 0.05*), while AST levels also decreased significantly in the insulin group (*p < 0.05*). No significant changes were observed in the control group. For pancreatic function markers, fasting insulin levels increased significantly in the control and insulin groups (*p < 0.05*), but not in the GLP-1RA group. Fasting C-peptide levels significantly increased in both the insulin (*p < 0.05*) and GLP-1RA (*p < 0.001*) groups, indicating potential enhancement of β-cell function. The homeostasis model assessment of insulin resistance (HOMA-IR) was significantly reduced in the insulin group (*p < 0.05*), but remained unchanged in the GLP-1RA and control groups. In contrast, the β-cell function index (HOMA-β) increased significantly across all three groups (*p < 0.001*), suggesting a general improvement in islet β-cell function following treatment. For DKD-related markers, both the insulin and GLP-1RA groups showed significant reductions in the urinary albumin-to-creatinine ratio (UACR) after treatment (*p < 0.001* for both), whereas no significant change was observed in the control group. In addition, both UACR and sCD36 levels were significantly decreased in the insulin and GLP-1RA groups (*p < 0.001*; **Figure 3**), with the greatest reduction observed in the GLP-1RA group, suggesting a potentially superior renoprotective effect of GLP-1RA therapy.

**Table 2B:** Comparison of parameter changes before and after treatment.

Variable	Treatment regimen (Control:0; Insulin:1; GLP-1: 2)	Before treatment	After treatment	Test value (t/Z)	*P*
FBG (mmoll)	0	7.47 ± 1.53	6.60 ± 1.01	5.039	*p<0.001*
	1	9.02 (7.89~10.32)	7.03 (6.20~7.75)	-6.936	*p<0.001*
	2	8.07 ± 1.52	7.10 ± 1.05	5.902	*p<0.001*
HbA1c	0	7.75 (6.98~8.70)	6.74 ± 0.97	-6.555	*p<0.001*
	1	9.35 (8.00~11.05)	7.16 ± 1.05	-7.324	*p<0.001*
	2	8.39 ± 1.44	6.72 ± 0.90	11.84	*p<0.001*
TG (mmoll)	0	1.55 (0.94~2.64)	1.33 (0.95~1.77)	-3.773	*p<0.001*
	1	1.76 (1.19~2.68)	1.40 (1.12~2.12)	-5.573	*p<0.001*
	2	1.91 (1.31~3.04)	1.37 (1.08~2.05)	-4.863	*p<0.001*
TC (mmoll)	0	4.12 (3.62~5.09)	4.08 ± 0.89	-1.804	*p=0.071*
	1	4.76 ± 1.31	4.22 ± 0.99	4.725	*p<0.001*
	2	4.75 ± 1.34	4.02 ± 0.92	4.883	*p<0.001*
LDL-C (mmol/l)	0	2.35 ± 0.72	1.97 (1.28~2.31)	-3.557	*p<0.001*
	1	2.72 ± 1.02	2.08 (1.55~2.87)	-5.08	*p<0.001*
	2	2.66 ± 1.06	2.13 ± 0.76	4.552	*p<0.001*
HDL-C (mmol/l)	0	1.17 ± 0.31	1.30 ± 0.30	-3.694	*p<0.001*
	1	1.10 ± 0.30	1.23 (1.05~1.33)	-3.968	*p<0.001*
	2	1.05 ± 0.25	1.20 (1.05~1.32)	-3.945	*p<0.001*
FFA (mmoll)	0	0.43 (0.29~0.62)	0.36 (0.29~0.44)	-3.722	*p<0.001*
	1	0.40 (0.28~0.49)	0.33 (0.21~0.44)	-2.797	*p<0.05*
	2	0.48 (0.35~0.60)	0.30 (0.24~0.38)	-4.319	*p<0.001*
UA (umoll)	0	344.50 (293.75~430.25)	306.00 (252.00~382.00)	-3.541	*p<0.001*
	1	336.45 ± 94.00	295.00 (246.00~370.00)	-4.283	*p<0.001*
	2	350.59 ± 88.88	288.00 (222.50~359.50)	-4.74	*p<0.001*
AST (UL)	0	17.00 (14.00~21.00)	17.24 ± 4.24	-1.337	*p=0.181*
	1	17.00 (15~23.00)	17.00 (14.00~21.00)	-2.041	*p<0.05*
	2	19.90 (15.00~29.00)	17.00 (14.00~21.50)	-3.914	*p<0.001*
ALT (UL)	0	17.50 (11.75~25.00)	18.00 (14.00~25.00)	-0.400	*p=0.689*
	1	18.50 (15.00~25.25)	20.00 (16.00~27.00)	-0.125	*p=0.90*
	2	21.50 (13.00~46.75)	21.00 (14.50~34.5)	-2.706	*p<0.05*
Fasting insulin (uIUmL)	0	3.57 (1.89~8.89)	3.67 (2.14~7.94)	-2.759	*p<0.05*
	1	7.57 (4.63~12.63)	9.51 (5.97~14.35)	-3.002	*p<0.05*
	2	12.15 (6.39~18.73)	13.95 ± 7.57	-1.414	*p=0.157*
Fasting C-peptide (ngml)	0	3.23 (2.03~7.90)	3.94 (1.98~7.61)	-1.715	*p=0.086*
	1	2.04 (1.33~2.85)	2.19 (1.46~3.46)	-1.969	*p<0.05*
	2	2.86 ± 1.45	3.41 ± 1.47	-5.546	*p<0.001*
UACR (mgg)	0	38.95 (33.22~76.89)	38.73 (32.51~71.93)	-0.322	*p=0.748*
	1	39.15 (35.06~53.62)	36.37 (27.40~40.68)	-5.286	*p<0.001*
	2	39.44 (35.52~48.50)	20.31 (14.33~32.86)	-6.567	*p<0.001*
HOMA-IR	0	1.25 (0.54~2.99)	1.15 (0.61~2.32)	-1.136	*p=0.256*
	1	3.07 (1.72~5.52)	3.04 (1.69~4.55)	-2.269	*p<0.05*
	2	4.07 (2.24~6.61)	4.27 (2.64~5.45)	-1.386	*p=0.166*
HOMA-β	0	20.71 (11.08~37.02)	26.07 (13.01~62.58)	-5.155	*p<0.001*
	1	28.09 (16.95~39.99)	58.24 (33.82~90.03)	-6.394	*p<0.001*
	2	56.54 (29.60~91.92)	78.87 ± 40.52	-4.008	*p<0.001*
sCD36	0	388.50 (342.55~466.10)	386.10 (323.60~471.30)	-0.335	*p=0.737*
	1	384.45 (349.28~440.33)	364.60 (279.10~394.10)	-5.352	*p<0.001*
	2	386.55 (354.85~437.23)	195.20 (160.45~314.75)	-6.567	*p<0.001*

FBG, Fasting Blood Glucose; BUN, Blood Urea Nitrogen; HbA1c, Glycated Hemoglobin; TG, Triglycerides; TC, Total Cholesterol; LDL-C, Low-Density Lipoprotein Cholesterol; HDL-C, High-Density Lipoprotein Cholesterol; FFA, Free Fatty Acids; UA, Uric Acid; ALT, Alanine Aminotransferase; AST, Aspartate Aminotransferase; UACR, Urinary Albumin-to-Creatinine Ratio; HOMA-IR, Homeostasis Model Assessment of Insulin Resistance; HOMA-β, Homeostasis Model Assessment of β-cell Functions; CD36, Soluble CD36.

### 3.4 Between-group comparison and covariate-adjusted analysis

This study further analyzed between-group differences in clinical indicators before and after treatment among the control, insulin, and GLP-1RA groups (**Table 3A**). Significant differences were observed among the groups in fasting blood glucose (FBG), glycated hemoglobin (HbA1c), fasting insulin, fasting C-peptide, homeostasis model assessment of insulin resistance (HOMA-IR), β-cell function index (HOMA-β), urinary albumin-to-creatinine ratio (UACR), and sCD36, with all *p < 0.05*.In parallel, within-group comparisons confirmed that all three groups experienced some degree of clinical improvement after treatment (**Table 2B**). Of particular interest, baseline levels of UACR and sCD36 did not differ significantly between the groups (*p > 0.05*); however, significant between-group differences were observed post-treatment (**Table 3A**), suggesting differential effects of the interventions on renal injury markers. Pairwise *post hoc* comparisons revealed that the GLP-1RA group exhibited significantly greater reductions in both UACR and sCD36 levels compared to the insulin and control groups (all *p < 0.05*), highlighting the superior renoprotective potential of GLP-1RA therapy in the management of DKD. Regarding metabolic indicators such as FBG, HbA1c, fasting insulin, C-peptide, HOMA-IR, and HOMA-β, significant baseline differences already existed among groups. These differences persisted in the post-treatment data and remained significant even in the change-from-baseline values (**Table 2B**), raising concerns that baseline imbalances may confound the interpretation of treatment effects. Given the statistically significant baseline differences in HbA1c, HOMA-IR, and fasting insulin levels across groups (p < 0.05), we performed ANCOVA to adjust for these imbalances when comparing post-treatment outcomes. This statistical approach allowed us to mitigate the influence of confounding variables and isolate the treatment effects more accurately. The ANCOVA results (**Table 3B**) showed that significant overall between-group differences remained for fasting insulin (F = 8.35, *p < 0.001*), HOMA-IR (F = 12.56, *p < 0.001*), and HOMA-β (F = 5.20, *p = 0.006*). However, subsequent pairwise comparisons using the least significant difference (LSD) method failed to detect statistically significant differences between individual treatment groups (all *p > 0.05*), indicating that although overall trends were present, definitive pairwise treatment effects could not be confirmed. In summary, the findings suggest that the different therapeutic regimens exert significantly different effects on DKD-related biomarkers, particularly UACR and sCD36, with GLP-1RA therapy demonstrating a potentially superior clinical benefit. For other metabolic indicators, such as fasting insulin, HOMA-IR, and HOMA-β, although overall group differences were observed, further studies with larger sample sizes are needed to confirm the specific intergroup effects. After adjusting for baseline HbA1c levels using ANCOVA, the between-group differences in post-treatment outcomes remained significant for sCD36 and UACR (p < 0.05), supporting the robustness of our findings.

**Table 3A:** Comparison of parameters between groups. TABLE 3(a)Between-group comparison of endpoint DKD parameters.

Endpoint parameters	Z	*P*
UACR(mg/g)		
Insulin group—GLP-1group	-4.667	*p<0.001*
Control group—GLP-1group	-5.137	*p<0.001*
Control group—Insulin group	-1.984	*p=0.047*
sCD36(ng/ml)		
Insulin group—GLP-1group	-5.221	*p<0.001*
Control group—GLP-1group	-5.607	*p<0.001*
Control group—Insulin group	-2.258	*p=0.024*

**Table 3B:** Between-group ANCOVA test.

Parameters	F	P
FBG	1.863	*p=0.158*
HbA1c	2.65	*p=0.073*
Fasting insulin	8.35	*p<0.001*
Fasting C-peptide	2.93	*p=0.056*
HOMA-IR	12.56	*p<0.001*
HOMA-β	5.2	*p<0.001*

FBG, Fasting Blood Glucose; HbA1c, Glycated Hemoglobin; HOMA-IR, Homeostasis Model Assessment of Insulin Resistance; HOMA-β, Homeostasis Model Assessment of β-cell.

### 3.5 Correlation and generalized linear regression analyses

Correlation analyses revealed that HbA1c, sCD36, and uric acid were significantly associated with UACR both before and after treatment (*p < 0.05*). To further investigate the strength of these associations, a generalized linear model (GLM) with a gamma distribution and log link function was employed to analyze baseline, post-treatment, and change-from-baseline values for HbA1c, uric acid, and sCD36. At baseline, only sCD36 was significantly associated with UACR (**Table 4**, regression coefficient = 0.007, Exp(B) = 1.007, 95% CI: 1.006–1.007, Wald χ² = 921.179, *p < 0.001*), indicating that elevated baseline sCD36 was strongly associated with increased risk of DKD progression. In contrast, baseline HbA1c (regression coefficient = 0.021, Exp(B) = 1.021, 95% CI: 0.994–1.049, *p = 0.129*) and uric acid (regression coefficient ≈ 0, Exp(B) = 1.000, 95% CI: 1.000–1.001, *p = 0.156*) were not significantly associated with UACR. After treatment, both sCD36 and uric acid remained significantly associated with UACR. Specifically, sCD36 had a regression coefficient of 0.006 (Exp(B) = 1.006, 95% CI: 1.005–1.006, Wald χ² = 728.521, *p < 0.001*), while uric acid had a coefficient of 0.001 (Exp(B) = 1.001, 95% CI: 1.000–1.001, Wald χ² = 8.009, *p < 0.05*). Post-treatment HbA1c was not significantly associated with UACR (regression coefficient = 0.036, Exp(B) = 1.037, 95% CI: 0.986–1.091, *p = 0.159*).Analysis of change values (post-treatment minus baseline) revealed that the change in HbA1c was significantly and negatively associated with UACR (regression coefficient = –0.410, Exp(B) = 0.664, 95% CI: 0.518–0.850, Wald χ² = 10.511, *p < 0.05*), suggesting that HbA1c reduction was closely related to improved DKD status. Additionally, the change in sCD36 was positively associated with the change in UACR (Exp(B) = 1.000, 95% CI: 0.997–1.002, *p<0.001*), indicating that a reduction in sCD36 was significantly correlated with DKD improvement. However, changes in uric acid were not significantly associated with UACR change (regression coefficient = 0.011, Exp(B) = 1.011, 95% CI: 1.005–1.017, Wald χ² = 12.440, *p>0.05*).

**Table 4:** Binary logistic regression analysis of early diabetic kidney disease patients and related parameters.

Parameters	Regression coefficient	Standard error	Wald χ²	*P*	Exp(B)	95%CI
Before treatment						
HbA1c	0.021	0.0139	2.309	*0.129*	1.021	(0.994, 1.049)
UA	0	0.0003	2.011	*0.156*	1.000	(1.000, 1.001)
sCD36	0.007	0.0002	921.179	*<0.001*	1.007	(1.006, 1.007)
After treatment						
HbA1c	0.036	0.0259	1.984	*0.159*	1.037	(0.986, 1.091)
UA	0.001	0.0003	8.009	*<0.05*	1.001	(1.000, 1.001)
sCD36	0.006	0.0002	728.521	*<0.001*	1.006	(1.005, 1.006)
Difference value						
HbA1c	-0.41	0.1263	10.511	*<0.05*	0.664	(0.518, 0.850)
sCD36	0	0.0012	0.029	*<0.001*	1.000	(0.997, 1.002)
UA	0.011	0.0031	12.44	*0.865*	1.011	(1.005, 1.017)

HbA1c, Glycated Hemoglobin; UA, Uric Acid; CD36, Soluble CD36.

## 4 Discussion

The molecular pathogenesis of DKD involves a complex interplay of lipotoxicity, oxidative stress, inflammation, and fibrosis. Among these, lipotoxicity plays a particularly prominent role in DKD progression. The central mechanism of lipotoxicity is the excessive accumulation and metabolic dysregulation of free fatty acids (FFAs). When FFAs accumulate in renal tubular epithelial cells, they induce mitochondrial dysfunction and impair ATP production, which in turn activates pro-inflammatory signaling pathways (5, 10).Oxidative stress represents another critical mechanism. Under hyperglycemic conditions and dysregulated lipid metabolism, excessive reactive oxygen species (ROS) are generated, disrupting the redox balance and exacerbating damage to both tubular and glomerular structures (11). Inflammation also plays a pivotal role in DKD progression, particularly through activation of the NLRP3 inflammasome. NLRP3 triggers caspase-1 cleavage, which activates interleukin-1β (IL-1β) and interleukin-18 (IL-18), both of which further compromise renal tissue integrity and promote inflammation and fibrosis in the tubules and glomeruli (12). Additionally, the Wnt/β-catenin signaling pathway has been closely linked to tubulointerstitial fibrosis. Its overactivation leads to abnormal accumulation of fibronectin (FN) and type IV collagen (COL4), thereby accelerating renal scarring and structural deterioration (6).

Numerous studies have confirmed a close association between CD36 and the progression of DKD. Elevated CD36 expression in DKD patients has been strongly correlated with renal structural damage, lipid accumulation, inflammation, oxidative stress imbalance, and the development of fibrosis (10, 13–15). CD36 contributes to renal injury through multiple and interrelated mechanisms: Inhibition of Mitochondrial Fatty Acid Oxidation (FAO): CD36 suppresses mitochondrial FAO, which leads to excessive production of mitochondrial reactive oxygen species (mtROS). This triggers the activation of the NLRP3 inflammasome and proinflammatory cytokines such as IL-1β, thereby aggravating renal inflammation and fibrosis (5, 6).Activation of the TRPC6/NFAT2 Signaling Pathway: CD36 has been shown to activate transient receptor potential channel 6 (TRPC6), leading to increased intracellular calcium concentration ([Ca^2+^]i) and subsequent activation of the NFAT2 signaling pathway. This process promotes transforming growth factor-beta1 (TGF-β1) expression and fibrotic responses in glomerular mesangial cells (HMCs). Furthermore, co-expression of CD36 and nuclear translocation of NFAT2 supports the notion that CD36 is a key regulator of palmitic acid (PA)-induced activation of the TRPC6/NFAT2 axis. This pathway links CD36 to T2DM-induced glomerular fibrosis, lipid metabolic disturbance, and calcium homeostasis dysregulation (3, 16).sCD36, the circulating form of CD36, is released into the bloodstream through proteolytic cleavage or shedding mechanisms. Although this study did not directly assess renal tissue CD36 levels, previous research using animal models and human biopsy specimens has demonstrated a strong positive correlation between circulating sCD36 concentrations and CD36 mRNA/protein expression in kidney tissues (7, 10).Building upon these findings, our clinical data further validate the relevance of sCD36 in reflecting CD36-mediated lipotoxic activity. Elevated sCD36 levels were closely associated with the severity of tubular lipotoxicity and inflammatory responses. As a dynamic biomarker, sCD36 may reflect lipid overload and oxidative stress in the kidney, thereby offering potential as a noninvasive indicator of renal injury. These observations are highly consistent with our current study results (6, 7).

Glucagon-like peptide-1 (GLP-1) is an incretin hormone that exerts its glucose-lowering effects primarily by stimulating glucose-dependent insulin secretion, inhibiting glucagon release, and regulating pancreatic β-cell proliferation, differentiation, and survival (17). GLP-1 receptors (GLP-1R) are widely expressed in various tissues, including the heart, lungs, liver, adipose tissue, and kidneys. Activation of GLP-1R not only improves dyslipidemia, insulin resistance, inflammatory responses, and hepatic steatosis, but also confers substantial cardiovascular and renal protective effects (18). Extensive research has elucidated several molecular pathways through which GLP-1RAs exert renoprotective effects:1, Inhibition of Sodium–Hydrogen Exchanger 3 (NHE3):GLP-1RAs inhibit NHE3 activity in the proximal tubules, thereby promoting natriuresis and diuresis. This reduces glomerular hyperfiltration and intraglomerular hypertension. In addition, GLP-1RAs suppress the expression of proinflammatory mediators such as NF-κB, IL-6, and TNF-α, attenuating inflammation-mediated glomerular injury. They also reduce renal ROS production, protect the integrity of tubular epithelial cells and podocytes, and reduce podocyte apoptosis and foot process effacement—ultimately strengthening the glomerular filtration barrier (19).2, Restoration of Oxidative Stress Balance: GLP-1 activation enhances the activity of antioxidant enzymes such as superoxide dismutase (SOD) and reduces levels of malondialdehyde (MDA), indicating significant antioxidant effects. GLP-1 also increases renal nicotinamide adenine dinucleotide (NAD^+^) levels and restores mitochondrial electron transport chain function, thereby improving overall antioxidant capacity. Furthermore, it lowers renal adenosine levels, mitigating inflammation within the kidney (20).3, Activation of AMPK and PPAR-α Signaling Pathways: GLP-1RAs activate the AMP-activated protein kinase (AMPK) and peroxisome proliferator-activated receptor-alpha (PPAR-α) signaling pathways, leading to reduced lipid peroxidation and endothelial injury. These effects alleviate inflammation and fibrosis in the glomerular basement membrane and contribute to preservation of the glomerular filtration barrier (21).

**Figure 4:**
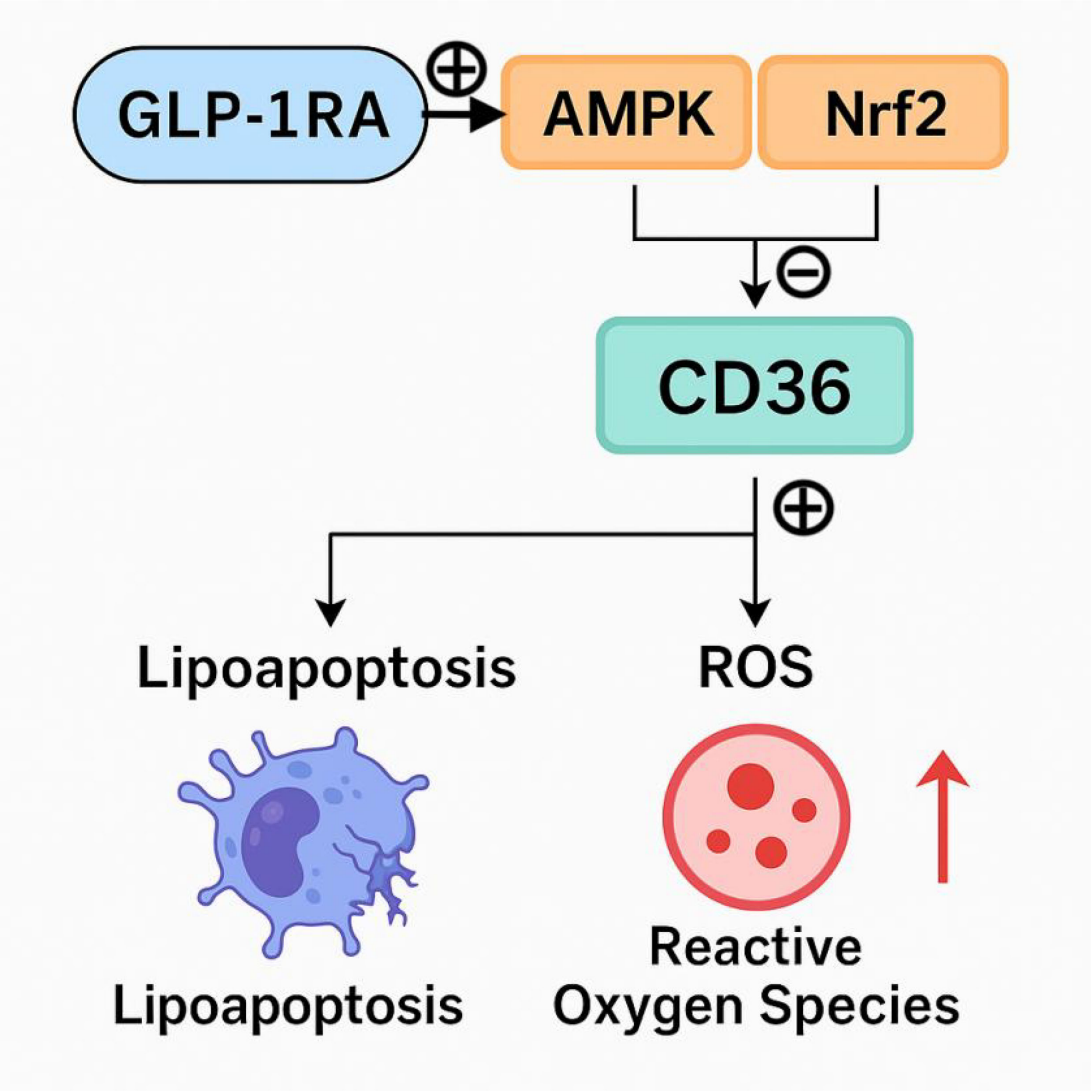
Potential mechanism of GLP-1 in alleviating diabetic kidney disease (DKD).

Our findings demonstrated a significant reduction in sCD36 levels in the GLP-1RA group compared to both the insulin and control groups. Although sCD36 also declined in the insulin group, this change may be primarily attributed to improved glycemic control and overall metabolic homeostasis, rather than a direct regulatory effect of insulin on CD36 expression. Notably, several previous studies suggest that GLP-1RAs may exert renoprotective effects beyond glycemic control by modulating CD36 through specific intracellular signaling pathways. Activation of AMP-activated protein kinase (AMPK) by GLP-1 has been shown to suppress high-glucose–induced metabolic reprogramming, downregulate CD36 expression, reduce mitochondrial reactive oxygen species (mtROS) production, inhibit NLRP3 inflammasome and IL-1β activation, and restore mitochondrial fatty acid oxidation (FAO) (5, 22). Collectively, these processes alleviate renal inflammation and fibrosis, thus enhancing renal protection. Additionally, *in vitro* studies have demonstrated that CD36 expression is markedly reduced in renal proximal tubular cells (RPTCs) lacking nuclear factor erythroid 2–related factor 2 (NRF2), with concomitant decreases in lipid accumulation and structural kidney injury (23, 24). Recent evidence also suggests that GLP-1RAs may activate the NRF2 signaling pathway, leading to upregulation of antioxidant defense genes and attenuation of oxidative stress and inflammation (25). These findings indicate that the beneficial effects of GLP-1RAs may involve more complex and multifaceted signaling cascades, including AMPK and NRF2 pathways (**Figure 4**). However, it is important to emphasize that our current study did not include molecular experiments such as gene or protein expression assays to directly investigate these signaling mechanisms. As such, the proposed link between GLP-1RAs, CD36 modulation, and renal protection remains speculative and is based on previously published literature. Although our findings suggest that GLP-1RAs may modulate CD36 levels and improve renal outcomes, the underlying molecular mechanisms—particularly the involvement of AMPK or NRF2 signaling pathways—remain hypothetical. Our study did not include direct assessment of gene or protein expression related to these pathways. The proposed mechanisms are derived from previously published *in vitro* and animal studies. Therefore, the suggested regulatory role of GLP-1RAs via AMPK/NRF2 signaling should be interpreted with caution and warrants further validation through mechanistic investigations in experimental models. We acknowledge that baseline differences in metabolic parameters, such as HbA1c, may influence treatment outcomes. Notably, sCD36 levels may also be affected by non-disease-related factors such as age, smoking, dietary intake, and platelet count. Although age was included as a covariate in our regression analysis, and the treatment effect of GLP-1RAs on sCD36 remained significant after adjustment, residual confounding from unmeasured variables cannot be ruled out. Particularly, the GLP-1RA group was significantly younger than the insulin and control groups, which may partially contribute to the observed sCD36 reduction. Future studies should incorporate a more comprehensive set of covariates and consider stratified analyses or propensity score matching to minimize bias. Furthermore, while prior evidence suggests that sCD36 levels do not differ markedly between diabetic and non-diabetic populations (26), its association with renal injury in DKD contexts remains an area of active investigation.

In summary, this study demonstrated a significant reduction in sCD36 levels among patients in the GLP-1RA treatment group, accompanied by a more pronounced improvement in the urinary albumin-to-creatinine ratio (UACR). These findings are consistent with previous literature (10) and suggest that sCD36 may serve not only as a promising biomarker for monitoring the progression of DKD, but also as a potential therapeutic target. However, the clinical progression of DKD is governed by complex molecular mechanisms. While GLP-1RAs have shown notable efficacy in alleviating renal injury, reducing UACR, and lowering sCD36 levels, the precise molecular pathways underlying these benefits remain incompletely understood. Further large-scale, high-quality clinical trials are needed to elucidate these mechanisms and to better define the role of GLP-1RAs in the treatment of DKD. Such efforts will ultimately help to refine therapeutic strategies and improve clinical outcomes for patients with DKD.

This study investigated the role of sCD36 in DKD and evaluated the impact of insulin and GLP-1RAs on disease progression. However, several limitations should be acknowledged:1, Sample Size and Duration: The sample size was relatively small, and the study duration was limited to 12 weeks. Long-term follow-up was not conducted, which limits the ability to assess the sustained effects of pharmacologic interventions on sCD36 levels and renal function. This may affect the generalizability of the findings and restrict conclusions regarding long-term outcomes. Moreover, while this study acknowledged the short treatment duration as a limitation, it did not comprehensively address the potential metabolic rebound effects following GLP-1RA discontinuation. Recent evidence suggests that withdrawal from GLP-1RA therapy may result in weight regain, increased adiposity, and reversal of metabolic improvements, including glycemic control and lipid metabolism. These rebound phenomena could also influence sCD36 levels and renal biomarkers such as UACR, thereby attenuating the durability of treatment benefits. This highlights the critical importance of treatment adherence and the need for long-term follow-up to evaluate the sustainability of GLP-1RA-induced improvements. Future clinical trials should incorporate extended monitoring periods and investigate strategies to maintain metabolic gains post-treatment cessation (27, 28). Consistent with previous findings, SS31, an antioxidant peptide, has been shown to downregulate CD36 expression and improve renal function in diabetic nephropathy, supporting the role of CD36 modulation in renoprotection (29). 2, Population Diversity: As a single-center observational cohort study, the results may be influenced by regional clinical practices and selection bias. The external validity of the findings requires cautious interpretation, particularly when applied to populations with diverse demographic and clinical characteristics.3, Limited Mechanistic Investigation: Although the study identified associations between sCD36 levels and DKD progression, the underlying molecular mechanisms—especially the regulatory effects of GLP-1RAs on CD36 expression and their renoprotective actions—remain insufficiently explored. Additional mechanistic studies are warranted.4, Scope of Intervention: Only two therapeutic strategies—insulin and GLP-1RAs—were investigated. The inclusion of other antidiabetic agents, such as sodium-glucose cotransporter 2 (SGLT2) inhibitors, could have provided a more comprehensive understanding of the effects of different treatments on sCD36 and DKD progression.5, Potential Confounding Factors: Although adjustments were made for key variables, unmeasured confounding factors such as dietary patterns and physical activity may have influenced the outcomes.6, It is important to acknowledge that this study utilized a non-randomized, observational cohort design, which introduces potential selection bias. Despite our use of ANCOVA to adjust for baseline differences in metabolic variables such as HbA1c and HOMA-IR, residual confounding cannot be completely excluded. Future randomized controlled trials with larger sample sizes are needed to confirm the causal relationship between GLP-1RA treatment and changes in sCD36 levels.7, Although sCD36 has been shown in previous studies to correlate with renal CD36 expression and injury in DKD models, it is important to note that sCD36 is not renal-specific. As a circulating molecule derived from multiple tissues—such as adipose tissue, macrophages, and endothelial cells—its systemic nature may limit its ability to precisely reflect kidney-specific CD36 activity. Therefore, the observed association between sCD36 and renal injury in this study should be interpreted with caution. Further studies incorporating renal biopsy specimens or urinary biomarkers (e.g., KIM-1, NGAL) are warranted to validate the renal specificity of sCD36 as a biomarker. Given these limitations, future studies should be designed as large-scale, multicenter, prospective trials with extended follow-up periods to further validate the dynamic role of sCD36 and its clinical utility as a prognostic biomarker in DKD.

## 5 Conclusion

This study is one of the first to systematically evaluate changes in soluble CD36 (sCD36) levels in response to GLP-1 receptor agonist (GLP-1RA) therapy based on real-world clinical data from patients with early-stage diabetic kidney disease (DKD). The results contribute novel insights into the potential role of sCD36 in DKD progression and therapeutic monitoring.1, we found that sCD36 levels were significantly reduced in the GLP-1RA group, and this reduction was positively correlated with improvement in urinary albumin-to-creatinine ratio (UACR), highlighting a strong association between sCD36 and renal function in DKD.2, based on prior literature, we propose that GLP-1RAs may exert renoprotective effects by modulating CD36-related molecular pathways, potentially reducing renal lipotoxicity and inflammation. However, the exact molecular mechanisms remain to be validated in future experimental studies.3, our findings support the potential of sCD36 as a noninvasive and dynamic biomarker for assessing DKD progression and treatment response. Given the study’s limitations—including a modest sample size, single-center design, and short observation period—future large-scale, multicenter, and long-term studies are warranted to confirm these associations and further explore the clinical utility of sCD36 and CD36-related pathways in the personalized management of DKD. Further molecular studies are warranted to confirm whether these pathways are indeed involved in the observed sCD36 modulation and renal outcomes.

## Data Availability

The original contributions presented in the study are included in the article/supplementary material. Further inquiries can be directed to the corresponding authors.
